# Comparison of immune modulation by TLR8 agonist vtx-2337 (motolimod) in cancer patients and healthy volunteers

**DOI:** 10.1186/2051-1426-2-S3-P165

**Published:** 2014-11-06

**Authors:** Greg Dietsch, Sam Whiting, Donald Northfelt, Ramesh Ramanathan, Peter Cohen, Kristi Manjarrez, Mona Newkirk, James Kyle Bryan, Robert Hershberg

**Affiliations:** 1 VentiRx Pharmaceuticals, Seattle, WA, USA; 2 Mayo Clinical Cancer Center - Research, Scottsdale, AZ, USA; 3 Virginia G. Piper Cancer Center (VGPCC) Clinical Trials Program at Scottsdale Healthcare, Scottsdale, AZ, USA; 4 Mayo Clinic in Arizona, Scottsdale, AZ, USA

## 

The potential benefits of stimulating a patient's immune system to fight cancer are profound. Historically, immunotherapy has been a successful treatment approach for some cancer patients, and recent advances in immunotherapy highlight that the immune system can generate durable tumor responses and cures. However, a concern regarding immunotherapy in cancer patients is the possibility that the immune response will be compromised by the underlying cancer and/or previous anticancer therapies. A promising approach in immunotherapy is targeting pathogen-associated molecular pattern receptors, such as the Toll-like receptors (TLRs), to activate the innate immune system and enhance development of tumor-directed adaptive immune responses. To assess whether cancer patients exhibit immune insufficiency in response to the potent and selective TLR8 agonist motolimod, we compared the pharmacokinetic and pharmacodynamic (PK/PD) relationship defined in a Phase 1 study of motolimod in cancer patients to the response in healthy volunteers.

Stimulation of healthy volunteer human PBMC by motolimod using the TruCulture^® ^system (Myriad-RBM) defined the breadth and magnitude of immune mediators induced by TLR8 activation in vitro. In a Phase 1 dose-escalation study in patients with advanced solid tumors, many of these defined mediators of TLR8 activation were increased in the plasma of cancer patients treated with motolimod. Notably, multiple biomarkers of immune activation, including G-CSF, MCP-1, and MIP1-β increased in a dose-dependent manner and correlated with increasing plasma levels of motolimod. A second Phase 1 study of motolimod in healthy volunteers provided an opportunity to compare and contrast the immune response generated by treatment with motolimod in healthy volunteers to that observed in cancer patients.

At comparable dose levels, the PK profile and overall exposure (AUC) to motolimod were similar for cancer patients and healthy volunteers. Motolimod induced the same repertoire of circulating cytokines and chemokines, indicative of TLR8 activation, in both populations. The magnitude of the mediator response in cancer patients was highly comparable to the response in healthy volunteers that received a similar dose.

In summary, advanced cancer and prior treatment with cytotoxic agents did not appear to blunt the response to motolimod based on the PK/PD relationship using predictive biomarkers. This comparison demonstrates that the immune system of cancer patients with advanced disease remains highly responsive to TLR8 activation by motolimod.

